# Supplementary material for: Disentangling snakebite dynamics in Colombia: How does rainfall and temperature drive snakebite temporal patterns?
Source: PLoS Negl Trop Dis. 2022 Mar 31;16(3):e0010270. doi: 10.1371/journal.pntd.0010270 (PMC8970366; doi:10.1371/journal.pntd.0010270)
Supplement: S1 Text — This section contains the development and explanation of the models. We used four models in our modeling framework to determine the association between climatic covariates and snakebite incidence, which are explained in Fig A. (DOCX) [file pntd.0010270.s001.docx]

**S1 Text. Compartmental models and B-splines.**

We based our models in the law of mass action, where the incidence will be proportional to the contacts between humans and venomous snakes and these contacts will be proportional to the multiplication of the abundance of both populations. This model can be seen in equation 1.

$$\begin{aligned} Incidence=\beta^{a}SV\#\left( 1 \right) \end{aligned}$$

Here, $\beta^{a}$ is the absolute contact rate between both populations, $S$ is the susceptible population, and $V$ will be the venomous snake population. Given that we don’t know how climate affects snakebite incidence, if it is via venomous snakes population dynamics ($V$) or via contact rate ($\beta^{*}$), we merged both in a new parameter $\beta$, which is what we denote an encounter frequency with venomous snakes. This parameter will depend on climate covariates ($C$). In addition, we added a Gaussian noise ($eps$) to this parameter, so our final model for incidence is shown in equation 2.

$$\begin{aligned} Incidence=e^{eps}\beta\left( C \right) S\#\left( 2 \right) \end{aligned}$$

To build the general epidemiological model, we assumed a susceptible population (*S*) and an “envenomed” population ($E$), where the flow from $S$ to $E$ will be the incidence. We assumed a constant recovery rate for $E$ ($\gamma$), and we assumed no mortality by snakebite. Thus, our general epidemiological model is shown in equation 3.

$$\begin{aligned} \frac{dS}{dt}=\gamma E-e^{eps}\beta C S; \frac{dE}{dt}=e^{eps}\beta\left( C \right) S-\gamma E \#\left( 3 \right) \end{aligned}$$

In this model, total population ($N$) will be equal to $S + E$, and it is constant. Thus, we can replace $S$ by $N-E$, and we can reduce our system to only one equation that is shown in equation 4.

$$\begin{aligned} \frac{dE}{dt}=e^{eps}\beta\left( C \right) \left( N-E \right)- \gamma E\#\left( 4 \right) \end{aligned}$$

Finally, we can discretize this equation to obtain the final general epidemiological model, which is shown in equation 5.

$$\begin{aligned} E_{n+1}={E_{n}+e}^{eps}\beta\left( C \right) \left( N_{n}-E_{n} \right)dt-\gamma E_{n}dt\#\left( 5 \right) \end{aligned}$$

For this general model, we first generated 2 models at a national scale to test if maximum temperature, minimum temperature, or rainfall drives snakebite incidence in the country. First, for Model 1 (Equation 6), the contact rate will be constant, thus climatic covariates do not modulate snakebite incidence. For Model 2 (Equation 7), the contact rate will depend on climatic covariate with a type III functional. We derived from model 2 three models: Model 2MXT where the climatic covariate is maximum temperature, Model 2MNT where the climatic covariate is minimum temperature, and Model 2R where the climatic covariate is rainfall.

$$\begin{aligned} MODEL 1: E_{n+1}={E_{n}+e}^{eps}\beta\left( N_{n}-E_{n} \right)dt-\gamma E_{n}dt\#\left( 6 \right) \end{aligned}$$

$$\begin{aligned} MODEL 2: E_{n+1}={E_{n}+e}^{eps}\beta^{*}\left( \frac{\left( C+\theta\right)^{2}}{K+\left( C+\theta\right)^{2}} \right) \left( N_{n}-E_{n} \right)dt-\gamma E_{n}dt\#\left( 7 \right) \end{aligned}$$

In this model, $\beta^{*}$ is proportionality constant which will set the average value of the contact rate, $\theta$ is an offset for the detrended and normalized rainfall, and $K$ is the constant for the slope of the functional response. Given that after adjusting Model2 to national data temperature did not modulate snakebite incidence, we did not account for this variable in the modelling scheme for every region. Thus, we only used rainfall as the climatic covariate at a regional scale.

After adjusting Model 1 and Model 2R, the modelling scheme will answer if rainfall modulates snakebite incidence. We looked for any rainfall-independent seasonality in regions where Model 1 adjusted better data than Model 2R (i.e., Rainfall does not drive snakebite incidence) by defining Model 3 (Equation 8). This model will assume that $\beta$ depends on only seasonality, which is a function that depends on 4 B-splines ($b_{i}$) (View S1.1). Finally, to answer if the seasonal component of rainfall (rainfall-driven seasonality) is the component of the signal that modulates snakebite incidence, we proposed model 4 (Equation 9). This model is equal than Model 2, but the climatic covariate is the rainfall-driven seasonality ($\bar{P}_{n}$).

$$\begin{aligned} MODEL 3: E_{n+1}={E_{n}+e}^{eps}\beta^{*}\left( \sum_{i=1}^{4} a_{i}b_{i} \right) \left( N_{n}-E_{n} \right)dt-\gamma E_{n}dt\#\left( 8 \right) \end{aligned}$$

In this model, $a_{i}$ is a lineal parameter which represent the strength of each seasonal component ($b_{i}$) in the contact rate.

$$\begin{aligned} MODEL 4: E_{n+1}={E_{n}+e}^{eps}\beta^{*}\left( \frac{\left( \bar{P}_{n}+\theta\right)^{2}}{K+\left( \bar{P}_{n}+\theta\right)^{2}} \right) \left( N_{n}-E_{n} \right)dt-\gamma E_{n}dt\#\left( 9 \right) \end{aligned}$$

The seasonal components, that depends on the B-splines ($b_{i}$), can be seen in the figure S1.1.


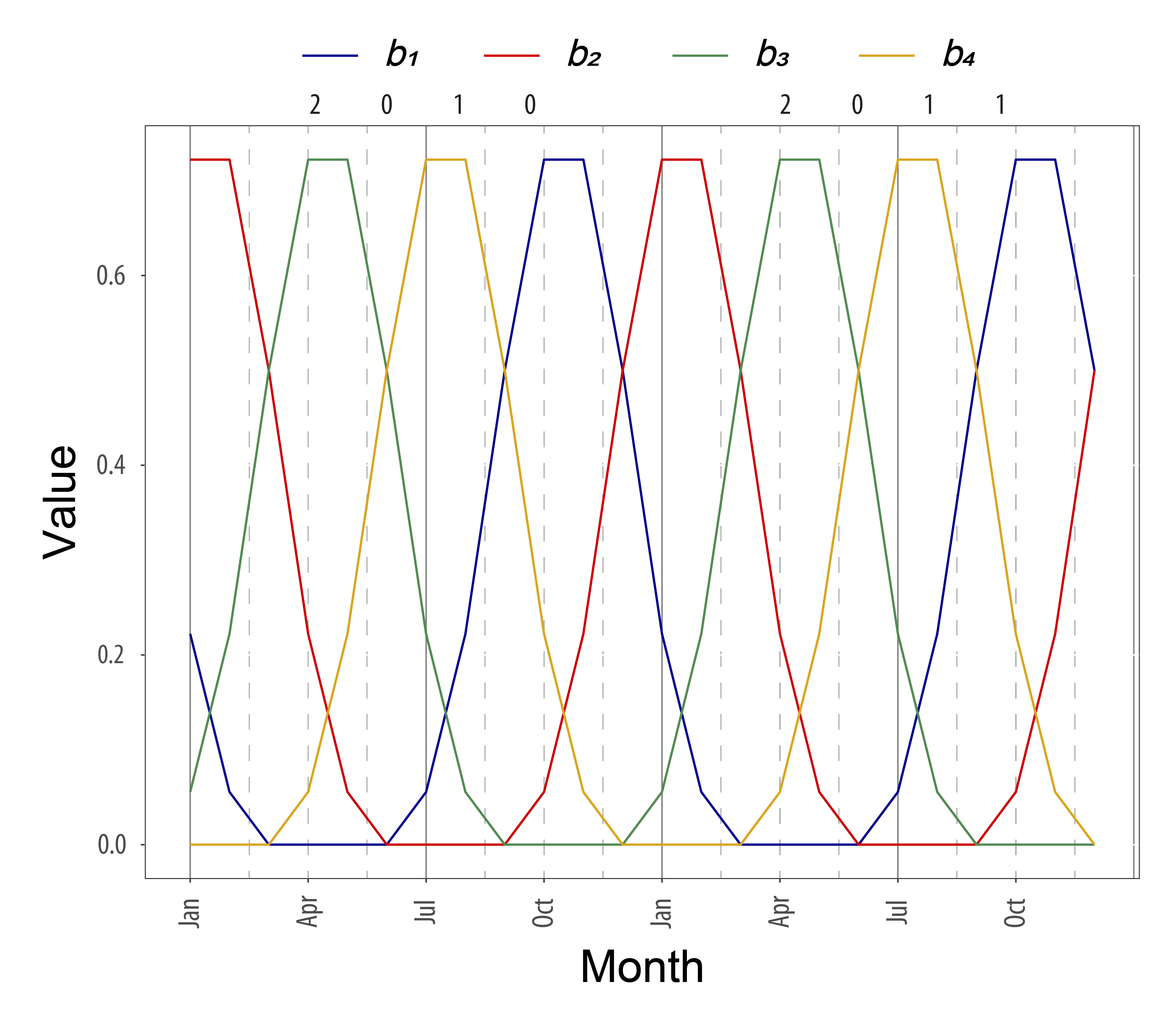


**Fig A. Seasonal components (B-splines) used in the proposed epidemiological models.**
