# Supplementary material for: Disentangling snakebite dynamics in Colombia: How does rainfall and temperature drive snakebite temporal patterns?
Source: PLoS Negl Trop Dis. 2022 Mar 31;16(3):e0010270. doi: 10.1371/journal.pntd.0010270 (PMC8970366; doi:10.1371/journal.pntd.0010270)
Supplement: S2 Text — We used a Poisson process to model data gathering, and then we used a particle filtering algorithm in two steps to adjust our modeling scheme to the data. This process is explained here. Fig A shows the results of the regions with similar rainfall patterns after clustering algorithm. Table A shows the confidence intervals for all parameters in all models after particle filtering algorithm. Fig B compares the adjusted encounter frequency for MODEL 1 between all regions, showing that regions where Bothrops atrox is distributed share the highest values. Table B shows seasonal parameters for rainfall in our defined regions and Fig C contains the seasonal distribution of rainfall over clusterized regions. (DOCX) [file pntd.0010270.s002.docx]

**S2 Text. Settings for parameter estimation and model selection.**

We modeled data recording as a Poisson process, with an average value described by the incidence’ model shown in S1. This model is shown in equation 10.

$$\begin{aligned} {Recorded incidence}_{i}\sim Poisson\left( \lambda=e^{eps}\beta\left( \bar{P} \right) \left( N-E \right) \right)\#\left( 10 \right) \end{aligned}$$

We performed a particle filtering algorithm with 2 steps. The specifications for the first step are:

- Number of particles: 100.
- Number of iterations: 100.
- Magnitude of the random walk perturbations: 0.02.
- Cooling fraction: 0.1.
- Cooling type: Hyperbolic.
- Particles to estimate likelihood for best combination of parameters: 100

Then, over the 10 best fittings for previous step, we performed a second search, defined with these specifications:

- Number of particles: 150.
- Number of iterations: 80.
- Magnitude of the random walk perturbations: 0.05.
- Cooling fraction: 0.1.
- Cooling type: Hyperbolic.
- Particles to estimate likelihood for best combination of parameters: 100

For model 2, 3 and 4 we did both steps to estimate all parameters. Then, we fixed the value for parameter $\beta^{*}$ at the value that maximizes the likelihood to reduce the confidence intervals for the other parameters, and we repeated both steps again.

This work was performed in r environment, using the package ‘pomp’ [1].

REFERENCES

1. King AA, Nguyen D, Ionides EL. Statistical inference for partially observed markov processes via the R package pomp. J Stat Softw [Internet]. 2016 Mar 25 [cited 2020 Mar 30];69:1–43. Available from:<http://arxiv.org/abs/1509.00503>

**Fig A. Clustered regions with similar rainfall patterns obtained after clustering algorithm.** Base map of national boundaries of Colombia was obtained from DIVA-GIS free spatial data (<https://www.diva-gis.org/datadown>). Shaded-relief base map for Colombia was obtained from Natural Earth free vector and raster maps (https://www.naturalearthdata.com/downloads/10m-raster-data/10m-shaded-relief/).

**Table A. Confidence intervals after parameter adjustment for all models in all regions.**

|  |  | **Parameter** | | | | | | |
| --- | --- | --- | --- | --- | --- | --- | --- | --- |
|  |  | ***β*** | ***K*** | ***θ*** | ***b_1_*** | ***b_2_*** | ***b_3_*** | ***b_4_*** |
| **MODEL 1** | **National** | 8.43e^-6^ - 8.99e^-6^ | - | - | - | - | - | - |
|  | **Region 1** | 2.29e^-5^ - 2.6e^-5^ | - | - | - | - | - | - |
|  | **Region 2** | 4.19e^-6^ - 4.58e^-6^ | - | - | - | - | - | - |
|  | **Region 3** | 2.57e^-5^ - 2.81e^-5^ | - | - | - | - | - | - |
|  | **Region 4** | 7.16e^-5^ - 8.42e^-5^ | - | - | - | - | - | - |
|  | **Region 5** | 3.58e^-5^ - 4.1e^-5^ | - | - | - | - | - | - |
|  | **Region 6** | 1.29e^-5^ - 1.42e^-5^ | - | - | - | - | - | - |
| **MODEL 2R** | **National** | 1.07e^-5^ | 0.3 - 1.1 | 0.7 - 2.2 | - | - | - | - |
|  | **Region 1** | 3.48e^-5^ | > 0.2 | 0.6 - 4.9 | - | - | - | - |
|  | **Region 2** | 4.4e^-6^ | > 0 | > 0.02 | - | - | - | - |
|  | **Region 3** | 4.25e^-5^ | 0.5 - 12.5 | 0.9 - 3.7 | - | - | - | - |
|  | **Region 4** | 8.17e^-5^ | > 0.01 | 2.3 - 6.01 | - | - | - | - |
|  | **Region 5** | 4.94e^-5^ | 0.08 - 0.8 | 0.3 - 1.2 | - | - | - | - |
|  | **Region 6** | 1.68e^-5^ | 0.2 - 0.5 | 0.09 - 0.6 | - | - | - | - |
| **MODEL 3** | **Region 1** | 2.6e^-5^ | - | - | 0.7 - 1.04 | 0.8 - 1.2 | 0.9 - 1.3 | 0.7 - 1.06 |
|  | **Region 2** | 4.4e^-6^ | - | - | 0.84 - 1.12 | 0.9 - 1.2 | 1.06 - 1.4 | 0.6 - 0.9 |
|  | **Region 4** | 1.01e^-4^ | - | - | > 0 | > 0 | > 0 | > 0 |
| **MODEL 4** | **National** | 1.19e^-5^ | 3.8 – 9.2 | 3.3 – 5.08 | - | - | - | - |
|  | **Region 3** | 5.78e^-5^ | 20.2 – 66.1 | 4.03 – 9.4 | - | - | - | - |
|  | **Region 5** | 5.82e^-5^ | 0.8 – 3.2 | 1.0 – 2.6 | - | - | - | - |
|  | **Region 6** | 3.04e^-5^ | 11.5 – 24.5 | 2.1 – 3.27 | - | - | - | - |

Confidence level: 95%.

* For MODEL 2R, MODEL 3 and MODEL 4 we fixed the parameter *β****^*^*** to the value that maximized the likelihood in a first search to reduce noise in the intervals.

**
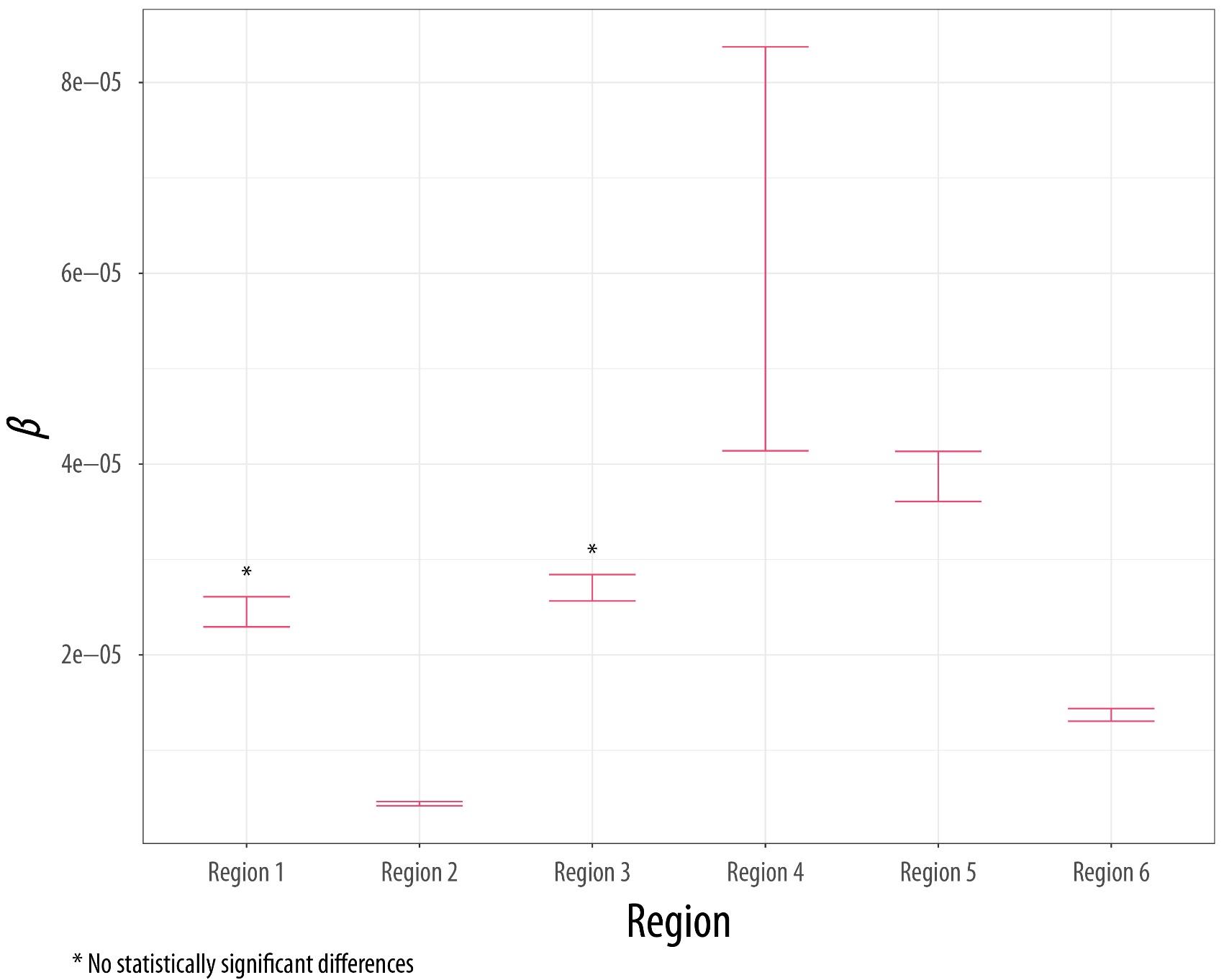
**

**Fig B. Likelihood confidence intervals over encounter frequency with venomous snakes after fitting MODEL 1 (No rainfall nor seasonality effect) to data.** Note that Region 4 and 5, where *Bothrops atrox* is distributed, share the highest values for this parameter.

**Table B. Coefficient of variation and strength of seasonality for precipitation in each region.**

|  |  |  |  |  |
| --- | --- | --- | --- | --- |
|  | **Region** | **Coefficient of variation (Precipitation)** | **Strength of seasonality (Precipitation)** |  |
|  | Region 1 | 0.512 | 0.429 |  |
|  | Region 2 | 0.447 | 0.546 |  |
|  | Region 3 | 0.594 | 0.684 |  |
|  | Region 4 | 0.324 | 0.517 |  |
|  | Region 5 | 0.596 | 0.855 |  |
|  | Region 6 | 0.670 | 0.727 |  |
|  |  |  |  |  |

**
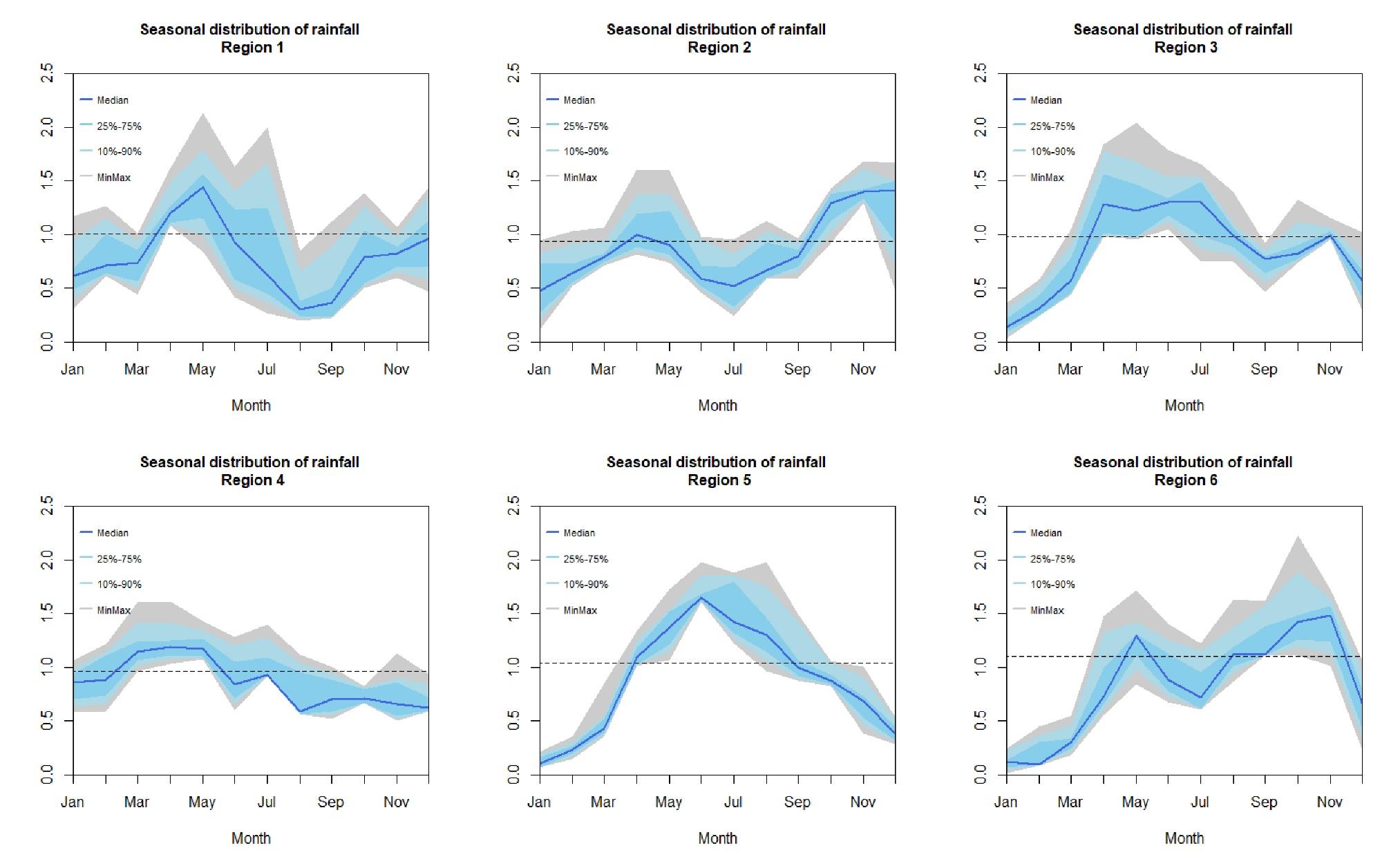
**

**Fig C. Seasonal distribution of rainfall over different regions of the country.** Note that Regions where rainfall drives snakebite incidence (Region 3, 5 and 6) share the lowest rainfall values during dry season.
