# Supplementary material for: Disentangling snakebite dynamics in Colombia: How does rainfall and temperature drive snakebite temporal patterns?
Source: PLoS Negl Trop Dis. 2022 Mar 31;16(3):e0010270. doi: 10.1371/journal.pntd.0010270 (PMC8970366; doi:10.1371/journal.pntd.0010270)
Supplement: Attachment — Submitted filename: Review.docx [file pntd.0010270.s003.docx]

Disentangling snakebite dynamics in Colombia: How does rainfall drive snakebite temporal patterns?

I must congratulate all the authors for carrying out this work. This is an important work in the field of snakebites. However, I have a few comments.

Major comments

- Line 115 : please explain what is a process-based model and provide details
- Line 190 – 195 : Not clear, please explain the aggregation procedure. Why did you aggregate and what is the resolution of data before and after aggregation?
- Model 4: Test if there is seasonality on snakebite incidence that is not explained by rainfall on areas where snakebite incidence depends on precipitation.
  - Rainfall is likely to have seasonal trends and can be easily confounded when both seasonality and rainfall is used simultaneously. Why authors did not evaluate the seasonalised rainfall (ie. rainfall anomaly after removing seasonal trend) in the analysis. Suggest to investigate this as well.
- Figures are difficult to understand, please increase the clarity and add explanation. Especially figure 5, need to provide details.

Minor comments

- Line 49 : please change as antivenom is not given instead of applied.
- Lines 51 – 52: but the spatial variation of this relationship has not been addressed in the literature
  - This statement is not correct
- Line 67 : replace medical care with allopathic medicine (as traditional medicine is also a medical care).
  - Please add Ediriweera et al (2017), “Health seeking behavior following snakebites in Sri Lanka: Results of an island wide community-based survey” as a reference to support lines 66 – 67.
- Lines 98 – 99: a recent study done in Sri Lanka has shown heterogeneity in the relationship between rainfall and snakebite incidence in different climatic zones. Therefore, pls update your statement with the below reference.
  - Ediriweera et al (2021), “Evaluating spatiotemporal dynamics of snakebite in Sri Lanka: Monthly incidence mapping from a national representative survey sample”.
