# Supplementary material for: Disentangling snakebite dynamics in Colombia: How does rainfall and temperature drive snakebite temporal patterns?
Source: PLoS Negl Trop Dis. 2022 Mar 31;16(3):e0010270. doi: 10.1371/journal.pntd.0010270 (PMC8970366; doi:10.1371/journal.pntd.0010270)
Supplement: Attachment — Submitted filename: Review-Snakebite Colombia.docx [file pntd.0010270.s004.docx]

**Reviewer comments for ‘Disentangling snakebite dynamics in Colombia: How does rainfall drive snakebite temporal patterns?’**

**General comments:**

This study provides important insight into the dynamics of snakebite in Colombia and provides an interesting methodology that is transferrable to other regions and can help with adequate snakebite management and antivenom distribution. Snakebite is a severely neglected tropical disease with an enormous global disease burden and research such as this is very important. In general I have no major concerns with the methodology or content of this study. However, there are some minor points I would like to see addressed.

There is some editing required to fix a variety of minor grammatical and wording issues. There are also some unnecessarily wordy sections. I’ve corrected many of these minor issues in my comments below (section Minor comments (grammar, spelling, wording, etc.)) but I recommend having a native English speaker look through the next draft before re-submission. Note, I’m not a native English speaker myself and, if in doubt, I have somebody look through my writing.

Regarding the content, please refer to my comments on content and context below.

**Comments on content and context:**

L67: and also because mortality number don’t accurately represent the additional burden caused by physical & mental disabilities following some snakebites

L81: you previously state that there are problems with extrapolation so you should explain here why your kind of extrapolation is different. For example geographically adjusted, fine scale extrapolation, or something similar that describes why your way of extrapolating is accounting better for the complexity of the situation than what is commonly done (i.e. what you criticized in the last paragraph).

L82-84: I think several authors would argue that they have used mechanistic epidemiological models for estimating temporal patterns in snakebite. Most notably Goldstein et al. 2021 (Integrating human behavior and snake ecology with agent-based models to predict snakebite in high-risk landscapes). In fact I’d be careful calling the models in the current study mechanistic. From my point of view a mechanistic model models specific processes to predict what snakebite incidence should be and then compares the predictions with real data. The current study is a correlative approach, where the correlation between rainfall and incidence is tested. It is partially mechanistic in that it tries to explain the ‘mechanism’ behind snakebite incidence trends (although rainfall alone is a very partial mechanism without accounting for other important drivers of snake activity such as temperature). However, it is not truly mechanistic in the form of a bottom up modelling approach. For this you would have to model how climate should, in theory, effect snake activity and abundance based on their known biology and then test if the predictions actually coincide with incidence. The approach in this paper is great, I’d just be careful with overstating the mechanistic nature of it, especially considering that there are studies in Sri Lanka that similarly correlate rainfall with snakebite incidence (Ediriweera et al. 2016; 2018) and that truly mechanistic snakebite models exist (Goldstein et al. 2021).

L250-259: I feel like it would have been worth testing the effect of other climate variables, such as e.g. temperature to explain the residual seasonality on top of rainfall driven patterns. Considering that you are trying to provide a mechanistic explanation of snakebite incidence, temperature is an important candidate variable and shouldn’t have been too difficult to include.

**Minor comments (grammar, spelling, wording, etc.):**

L31: ‘forcing’ is not the best word here, maybe ‘affecting’ or ‘driving’.

L34: delete ‘other’

L50: ‘where’ does not sound right here, do you mean ‘because’. Change typo ‘stablished’ to ‘established’

L53: delete ‘have’

L58: Replace ‘this’ with ‘our’

L59: delete ‘it’

L70: change risk’ to risk’s

L72: change ‘disease’ monitor tolls’ to ‘disease monitoring tolls’

L73: The problem is not the complexity of the extrapolation but the simplification of the extrapolations as compared to the complexity of reality. Reword.

L86: change ‘snakes’ species’ to ‘snake species’

L86: change ‘which often is poor’ to ‘which is often poor’.

L86: change ‘In neotropics’ to ‘In the Neotropics’

L87-88: change ‘which is distributed throughout the south of Mexico to the North of Argentina’ to which is distributed broadly from southern Mexico to northern Argentina’

L88: delete ‘the’ before envenomings

L89-91: too wordy, just say ‘This genus is viviparous with a clutch size of xxxx-xxxx offspring, which are born during the rainy season in many species.’

L92: change ‘venomous snakes’ populations’ to ‘venomous snake population’

L94: change ’resulting in an increase 94 in snakebite risk too’ to ‘resulting in an additional increase in snakebite risk’

L97: delete ‘in both countries’

L98: change ‘pattern’ to ‘patterns’

L100: change ‘Colombia becomes’ to ‘Colombia is’

L101 and throughout: I recommend changing ‘rainfall seasonal patterns’ to ‘seasonal rainfall patterns’

L104: delete ‘have’

L114: ‘aggregate municipality reported the incidence to reduce its noise’ This needs rewording, I’m not sure what you mean. Do you mean you aggregated individual municipalities’ reported data within each climate region to reduce noise in the data caused by different climate patterns?

L116: delete ‘a’ before likelihood

L129: change ‘the third model’ to ‘a third model’

L135-136: change ‘which model fit better the data’ to ‘the model with best fit’

L150 ‘The details and settings that we used to perform this estimation can be seen in S2.’ Is very wordy. You could just add a bracket saying ‘(see S2 for details)’

L152: change ‘likelihood were’ to ‘likelihood was’

L161-162: change ‘model 1, model 2, and model 4’ to ‘models 1, 2, and 4’ and change ‘and model 1 and model 3 too’ to ‘and so are models 1 and 3’

L162: please state the version of R you used here

L188L capitalize ‘r’, i.e. ‘R environment’ throughout the manuscript

L237: change ‘he becomes’ to ‘they become’ (gender neutral)

L251: change ‘maximizes a likelihood’ to just ‘maximizes likelihood’

L255: change ‘account’ to ‘accounts’

L257: change ‘explained better the data’ to ‘explained the data better’

L365: change ‘give birth neonates’ to ‘give birth to neonates’

L377: wouldn’t floods increase the area shared by snakes and humans, i.e. ring snakes closer to humans?

L391: change ‘disease’ burden’ to ‘disease burden’ or ‘burden of disease’
