# Supplementary material for: Disentangling snakebite dynamics in Colombia: How does rainfall and temperature drive snakebite temporal patterns?
Source: PLoS Negl Trop Dis. 2022 Mar 31;16(3):e0010270. doi: 10.1371/journal.pntd.0010270 (PMC8970366; doi:10.1371/journal.pntd.0010270)
Supplement: Attachment — Submitted filename: Response to reviewers.docx [file pntd.0010270.s005.docx]

**Manuscript PNTD-D-21-0130: Disentangling snakebite dynamics in Colombia: How does rainfall drive snakebite temporal patterns?**

**Point-by-point response to reviewers’ comments**

**Reviewer #1:**

**1. I only have two problems with methods. First is that, while methods are robust and novel in the field, I do not think that resulting models are epidemiological because they statistically estimate one parameter, labelled frequency of contact, as a function of rainfall. The authors try and convince the reader that because the estimated parameter is the frequency of contact, their models do not suffer from the correlation-causation ambiguity, although in essence that parameter is not different from previous estimates of incidence as a function of the environment. Therefore I suggest that such a description of their results should be avoided. I think then that the authors should seek to convince us that they have found a way to separate different sources of spatio-temporal variability of snakebite risk, which is on its own a very relevant development.**

We appreciate the interest of the referee in our work and the constructive comments. Therefore, we restructured the manuscript to address this critical point, and to change our scope from an epidemiological model to a compartmental model. The structure of our model is based on the scheme depicted in Figure 1, where each compartment represents a different population, and the flow from susceptible population to envenomed population is the new cases that occurs in a determined timestep. This flow is the incidence, which we used to calibrate our model with data.

Given that we didn’t model the snakes’ population, we removed the epidemiological term of our modeling scheme, and removed that idea our models do not suffer from the correlation-causation ambiguity. We stated clearly that this first step still has this ambiguity (Lines 635-636), and we changed the discussion to address the importance of our work: How to separate these sources of spatio-temporal variability. Finally, we added in the discussion the importance of including snakes’ biology to generate robust epidemiological models to avoid that correlation-causation ambiguity, and that our approach is a preliminary step to reach this objective.

**2. The second issue is related to data aggregation, in the conversion of municipalities from vector to raster, because vector data may have polygons larger than pixels, so when a polygon is converted to smaller pixels, all the pixels within a particular polygon will have the same population value. When these values are aggregated the resulting pixels will indicate a population size larger than it should be. Currently there is no information on whether this taken care of, or if it was considered, or if it actually is not a relevant issue and why.**

Thanks for noticing this issue. After re-reading the paper, we noted that this part in specific was not well-written. We considered this issue, so when we rasterized incidence, we divided the incidence value in each polygon by the number of pixels contained in it. So, the rasterized incidence is in fact a map of incidence per pixel. Therefore, the result of the sum of the pixels for each one of our six regions was not inflated. In fact, after performing the process, national incidence keeps constant. We also did the same procedure for the population. We are aware that we did not clarify this step; thus, we included it in methodology (Lines 196-208) a deeper explanation for this process. Also, we included a limitation of this process: It assumes that incidence and population distribution are homogeneous in each municipality, thus each pixel will contain the same value of incidence and population per pixel value.

**3. Another minor issue I have with methods is related to writing. There is a tendency to use jargon in places where we need to understand what authors did, for instance line 182 is very difficult to make sense, not only because it contains very technical terms, but because there are quantities mentioned of which there are no details given. Also, there is a lot of room for confusion in the use of rainfall and seasonality, because in occasions rainfall means seasonality, and viceversa.**

Thanks for the comment; we detected that in several parts of the text. We changed these technical terms throughout the manuscript, and we rewrote the paragraph about clustering. First, we clarified that reducing resolution in a factor of 12 was done by increasing pixel area 12 times. Thus, we included the resulting area of each pixel after this procedure to clarify the idea (L 177-193).

The comments also highlighted a problem in our manuscript had: The confusion about our model’s different kinds of seasonality. By suggestion of referee 2, we changed the last model (Model 4). This model had a component modulated by rainfall and another component by the seasonal b-splines. Given that these b-splines are flexible, they also represented the seasonality of rainfall, and they did not add any valuable information to our model. Therefore, we proposed a new model 4, which only uses the seasonal component of rainfall signal to detect if this rainfall-driven seasonality modulates snakebite incidence.

Finally, following the reviewer’s advice, we have changed all the seasonality terms in the paper to clarify the source of this seasonality. We have a rainfall-independent seasonality (Which we tested using Model 3 in areas where rainfall did not drive incidence) and rainfall-driven seasonality (Which we tested using this new Model 4, which uses only the seasonal component of rainfall (rainfall-driven seasonality) as covariate). Thanks to this, the manuscript is now easier to understand with more succinct ideas.

**4. Figures are all of very good quality, but captions should contain more succinct and understandable information. For instance, very few of us know how to interpret the "cross-wavelet" subfigure in Fig 1, so I don't know what's its relevance or what aspect of the data it explains, or describes, or which one of the phenomena in question it refers to. What do we learn about snakebites from this particular figure?**

Thanks for noticing this redundancy in the figures and our effort in making good quality figures. We modified all figures because some were not clear. For example, we redesigned figure 1 to show only information about our compartmental model because the cross-wavelet subfigure did not show any additional information. In addition, we included more succinct information in the captions of all figures to make them easier to understand. Figure 2 now contains the results of applying our modeling scheme to national data with rainfall-driven seasonality, maximum temperature, and minimum temperature. By suggestion of referee #3, we included temperature as a covariate to test its capacity modulating snakebite incidence in the country. Figure 3 shows the results of our modeling scheme for regions where rainfall did not drive snakebite incidence, and Figure 4 shows the results for regions where rainfall drive snakebite incidence. By performing these changes, figures are now more explainable, and the captions help to understand them better.

**5.** **The only major concern I have is with the narrative of their epidemiological models no having the causality problem one encounters with statistical models. This issue reappears in an important way in the conclusions.**

Thanks for pointing this out; we were unaware of it, but it is crucial in our research. We restructured our discussion and conclusions to remove this wrong narrative, and we changed epidemiological models by compartmental models, which we defined in the introduction (Lines 89 - 94). We clarified in conclusions that the strength of our approach with compartmental modeling is that they can be extrapolated easier than statistical models because, in our models, the incidence is represented by a flow between compartments where each parameter has biological meaning. We removed the causality problem for all our narratives because our approach can still be affected. In addition, in discussion (Lines 606-610), we explained better the difference between parameters in compartmental models and statistical models: Parameters have biological meaning in compartmental models, while in statistical models’ theses values are general. Finally, we included an explicit sentence (Lines 610 - 612) that explains how to improve our compartmental models by including venomous snakes’ population dynamics data to construct robust epidemiological models that can overcome the correlation-causation ambiguity.

**6. There are parts of the manuscript that need rephrasing, especially in methods.**

**Introduction**

**Line 73. I’d say, it’s strictly due to extrapolation and the process complexity that such models hide.**

We appreciate the advice and include it in this line. The statistical models hide the complexity of the reality, while the compartmental models try to represent it by representing populations in compartments and defining the flows between these compartments (Lines 84 – 86).

**Line 74. disease spread, not diseases’ spread.**

We changed this mistake.

**Line 75. Models in NTDs have been mainly used for endemic dynamics, not spread as in epidemic invasion, so i’d rephrase the previous sentence to reflect that characteristic of NTDs, it’s transmission rather than spread.**

Thanks for noticing this mistake. We were talking about this transmission rather than spread. We changed this line to overcome this problem (Line 88).

**Line 89. This group is viviparous... each female gives birth to several fully developed newborns.**

By suggestion of referee 3, we changed this line and included information about clutch size. This line became less wordy thanks to this new information (Lines 110 - 113).

**Line 100. Colombia is an ideal setting...**

We changed it. Thanks for your suggestion.

**Materials and methods**

**Line 110. That accounts for the role of climate. A model by itself, I beg to disagree, cannot “understand”.**

We changed it. Thanks for your suggestion.

**Line 114. municipality-reported incidence, remove “the” before incidence.**

We changed it. Thanks for your suggestion.

**Line 132. What does “ones” refer to? Does it mean that you added beta-estimates from both models?  Also, I don’t really follow the sentence “was only used on clusters...” does it mean that you only used model 4 on certain areas where the rainfall signal is stronger?**

We changed this sentence by the suggestion of referee 2. Model 4 now is a new model that uses only the seasonal rainfall component (Rainfall-driven seasonality), so this line was erased.

On the other hand, we noticed that our explanation of the modeling scheme was vague, so we included explicitly when and why we used Model 3 and Model 4: We used Model 3 to test if snakebite incidence is seasonal in regions where rainfall did not explain this variable, thus testing if there is any rainfall-independent seasonality in these regions. On the other hand, Model 4 tests if the seasonal component of rainfall (rainfall-driven seasonality) is the component of rainfall that modulates snakebite incidence. Thus, we only used Model 4 in regions where rainfall drives snakebite incidence (Model 2R adjusted better data than Model 1) (Lines 257 - 266).

**Line 140. Please define E before first mention. A general description of the model as is SIR would be useful. I also think your paper will benefit a lot from having a diagram that shows geometrically the difference between rainfall models.**

Thanks for the comment; we have noted that our methodological framework improved a lot by including it. We defined our models in an early section in methodology, and we did a deeper explanation of our model in supplementary materials. We also detailed in this supplementary section how the model was formulated.

Finally, we present in Figure 1 the compartment diagram of the general model. Given that the only change between models is the association between the encounter frequency *β* and the climatic covariates, this diagram is constant.

**Line 150. Suggest: parameter combinations, instead of “parameters combination”**

We changed it. Thanks for your suggestion.

**Line 152. Suggest: if model likelihoods were significantly different between each other**

We changed this part of the manuscript because we included different data as covariates: We used temperature for national data (Model 2MXT and Model 2MNT), and we changed the climatic covariate in Model 4 (Rainfall-driven seasonality). Thus, we use the Akaike information criterion (AIC) to compare models and select the best model representing data.

**Line 158. Suggest: a threshold of significance between likelihood differences.**

We changed this part of the manuscript because we are using AIC by suggestion of other referees.

**Line 162. Suggest: To fit models we used the pomp R package.**

We changed it. Thanks for your suggestion

**Line 182. By a factor of 12 what? Did you aggregate 12x12 pixels? Pixels around a 12 km radius?**

Thanks for noticing this mistake. Our idea was not evident in the manuscript. Now we have included the aggregation process explicitly. We included that we decrease resolution by increasing the area of each pixel by a factor of 12; thus, the new pixels are 12 times bigger than the original ones (Lines 179 - 181).

**Line 183. I think it’s easier to describe what the clustering procedure did, rather than list the names of the technics. While not being familiar with the methods, it’s very difficult to understand what you did.**

We appreciate your suggestion because now this part is easier to understand. We specified how does the algorithm work before describing the technical details (Lines 181 - 183).

**Line 183 Between 2 and 10 what? In general this paragraph is very difficult to understand, I get the feeling that it is about summarizing precipitation data in a way that best explains snakebite incidence data, but since it mainly contains very technical terms it’s hard to make sense out of it.**

Thanks for the suggestion. We did not notice that this paragraph was wordy and roughly technical. Therefore, we restructured the whole paragraph to introduce the aim of this clustering procedure (We wanted to define regions with similar rainfall patterns to adjust our modeling scheme in each region). In addition, to perform this clustering algorithm, it is needed to determine the number of clusters. Given that we did not know the optimal number of clusters, we applied the algorithm to divide the country between 2 and 10 regions, and then we computed the performance indexes to select the optimal number of clusters. This is now clearly defined in the paragraph (Lines 185 - 191).

**Lines 190-192. Are municipality and area synonims here?  How do these relate to “clusters”. I get the impression that you’re describing two ways of aggregating data, but it’s not clear at all.**

Thanks for noticing this grammar issue in the manuscript. We identified two issues: 1. We used clusters, areas, and regions to refer to the regions with similar rainfall patterns that we defined in our clustering procedure. Thus, we changed all these words by region. 2. We only aggregated data from municipalities to regions with similar rainfall patterns. In the paragraph of lines 195 - 208, we explained how we defined our regions, and in this paragraph, we explain how we converted data from municipality-scale to regional-scale. First, we averaged the rainfall for each region based on the rainfall rasters, and for incidence and population, we rasterized the municipality-based data (Polygons) and divided it by the number of pixels of each municipality. Afterwards, we summed the pixels of incidence and population per pixel for each region to obtain the total number of persons and cases in each of our regions with similar rainfall patterns. We restructured the paragraph to explain this procedure better.

**Results**

**Lines 201-205. This should be in methods section.**

Thanks for catching this. We have relocated this part in methods section (Lines 165 – 167).

**Line 206. Does “estimated seasonality” mean something like seasonal periods? So 6 months means two annual incidence “peaks” whilst 12 monts means one seasonal peak? Please clarify, because it is the whole point of the paper and should be spelled as clearly as possible in terms that non-modellers can understand.**

We relocated this part to the discussion section (Lines 510 - 515). Instead of using seasonality to refer to these periods, we made it simpler by defining the peaks of snakebite incidence that we found with our modeling scheme.

**Figure 1. I think the cross-wavelet figure and cross correlation function need further explanation. How do these sub-figures related to the biological phenomenon of snakebite? How can these subfigures be interpreted and what do they reflect about the de-trended data? Are they relevant to your main results?**

Thank you for seeing this redundancy in the figures. We changed all figures to make them more understandable, and we removed figures that were not relevant to our main results.

**Line 224. Figure 2 caption: models fitted to each region.**

We changed all the figures, so the captions are new. Thank you for the correction.

**Line 226. Figure 2 caption: indices instead of indexes.**

We changed all the figures, so the captions are new. Thank you for the correction.

**Line 237. so the person becomes... No need to assume any particular gender!**

We changed all the figures, so the captions are new. Thank you for this correction, and we agree with you: There is no need to assume a particular gender.

**Figure 2 caption. I think the use of seasonality and rainfall is extremely confusing, are  you saying that seasonality is a process independent of rainfall? This should be made clearerer and probably using different terms such as rainfall-driven seasonality vs rainfall-independent seasonality. I now see how the use of these terms relate to the model (4) but it can be made less confusing from the start if you use more informative names.**

Thank you for the suggestion. This suggestion coupled with the others referee suggestion made us to change Model 4 to reduce the confusion between different types of seasonality. We want to acknowledge you for your suggestion of terms, by including them in the paper (We only talk of rainfall-driven and rainfall-independent seasonality in the new manuscript) the redaction and understanding of the text improved a lot. We appreciate that a lot.

**Lines 250-253. This is just repetition from methods, so I think it can be removed unless you want to do a shorter recap.**

Thank you for your suggestion, we removed it.

**Lines 256-259. The use of the term national-aggregated data adds another layer of confusion here. I don’t know if it refers to the entire country’s incidence or to the particular clusters where model 4 was the “best”. Also, I think you should say nationally-aggregated data, unless I’m missing something.**

Thank you for noticing this confusion in the paper. When we talk about national-aggregated data we were just talking about national data. We changed it in the whole paper to remove this additional layer of confusion, and the paper became more understandable.

**Lines 278-280. We found that in three regions .... rainfall does not explain incidence.**

Thank you for your suggestion, we changed it.

**Line 281. in region 5, there is a stronger relationship between snakebite and rainfall…**

Thank you for your suggestion, we removed this part because we changed Model 4, and now this model is the best for all regions where rainfall drives snakebite incidence.

**Figure 3 caption (lines 287-303). Please fix the same aspect about other seasonality sources commented above.**

Thank you for your suggestion. Given that we changed figures, we changed the whole captions. Even so, we included the previously addressed seasonality confusion in the whole paper, where we only use two sources of seasonality: Rainfall-independent seasonality (Model 3) and rainfall-driven seasonality (Model 4).

**Line 299. precipitation drives snakebite strongly (at the end)**

Thank you for your suggestion. Given that we changed figures, we changed the whole captions.

**Line 303. This sounds like only region 5 has seasonal snakebites, which is why you need to make a clearer use of the terms rainfall and seasonality.**

Thank you for your suggestion. Given that we changed figures, we changed the whole captions.

**Lines 304-312. This paragraph needs a topic sentence.**

Thank you for your suggestion. By changing Model 4, we removed parts of this paragraph. Then, we included a topic sentence to this paragraph: This paragraph talks about how parameter’s confidence intervals affect the association between rainfall and snakebite incidence (Lines 441 – 447). The part of the paragraph where we stated rainfall as a limiting factor was moved to discussion (Lines 544 - 548).

**Discussion**

**Line 314. … the role of rainfall on snakebite seasonality.. (this needs more consistent use and clarification).**

Thank you for your suggestion. We clarified this part by specifying the role of climatic covariates modulating snakebite temporal patterns to avoid confusing the reader with seasonality.

**Line 342. Again, seasonality…**

Thank you for noticing this recurring problem. We changed this part by including the specific terms rainfall-independent and rainfall-driven seasonality.

**Line 344. By a larger rainfall pattern do you mean that the difference between wet and dry seasons is very large?**

Thank you for noticing this problem. Given that we restructured this paragraph by suggestion of the other referees, we removed this part.

**Lines 347-349 Suggestion: incidence and rainfall in Colombia is mediated via snakes’ activity… (delete what’s in between sentences)**

Thank your for your suggestion. In this part we included our hypothesis of rainfall acting as a limiting factor in snakebite dynamics. Thus, we restructured this paragraph and removed that part.

**Line 395. Same as per my comment above.**

Thank you for noticing this recurring problem. We changed this part by including the specific term rainfall-independent seasonality.

**Line 414. “undertaken”, instead of “done”.**

Thank you for your suggestion, we have changed it.

**Reviewer #2:**

**General comments:**

**1. Figures are difficult to understand, please increase the clarity and add explanation. Especially figure 5, need to provide details.**

We changed all figures and removed redundant information such as the cross-wavelet by the suggestion of referee 1. In addition, we changed the captions to include more information and a clear explanation of the figures.

**Specific comments:**

**2. Line 115 : please explain what is a process-based model and provide details**

Thanks for the suggestion. We changed the description of our model to a modeling scheme based on compartmental models as a suggestion of referee 3, who recommended us not to use that definition of the process-based model.

**3. Model 4: Test if there is seasonality on snakebite incidence that is not explained by rainfall on areas where snakebite incidence depends on precipitation.**

**Rainfall is likely to have seasonal trends and can be easily confounded when both seasonality and rainfall is used simultaneously.  Why authors did not evaluate the seasonalised rainfall (ie. rainfall anomaly after removing seasonal trend) in the analysis. Suggest to investigate this as well.**

We appreciated this recommendation because it changed several things in the article and made it more concrete and robust. Thanks to these constructive comments, the quality of the paper increased. After discussing this, we first tested the rainfall anomaly by removing the seasonality of the raw signal. Using this anomaly as a covariate, the models did not adjust the data well, and Model 1 (Null model without any covariate) was the one that adjusted better data. Thus, we thought that the seasonal component of rainfall was the driver of snakebite incidence. Next, we changed Model 4 because these B-splines confounded rainfall seasonality, and it was not adequate to use both signals simultaneously. Thus, the new Model 4 has the same architecture as Model 2R (Lines 257 – 259), but it uses as covariate the seasonal component of rainfall (By suggestion of referee one, we named it rainfall-driven seasonality).

Our new modeling scheme is the following: First, to test if rainfall drives snakebite incidence, we used Model 1 (Model without covariates) and compared it with Model 2R (Model with rainfall as a covariate). Then, in regions where Model 1 explained better data (Thus rainfall does not drive snakebite incidence), we looked for any rainfall-independent seasonality using Model 3 with seasonal B-splines. On the other hand, in regions where Model 2R fit better the data, we test with Model 4 if the seasonal component of this rainfall (Rainfall-driven seasonality) is the driver of snakebite incidence (Lines 293- 305). After performing these changes, we noted that for all regions where rainfall drives snakebite incidence, the seasonal component of this signal was the driver behind incidence temporal patterns.

**Minor comments**

**Line 49: please change as antivenom is not given instead of applied.**

We changed it by administered.

**Lines 51 – 52: but the spatial variation of this relationship has not been addressed in the literature: This statement is not correct.**

Thanks for noticing this. After re-reading the manuscript, we understood that we did not explain ourselves well; thus, the idea that we wanted to express here was difficult to understand. The spatial association between rainfall and snakebite incidence has been addressed in studies (Such as Chavez et al., 2015, and the studies of Yañez-Arenas et al., with snakes’ distribution, snakebite risk, and climate change). On the other hand, it is also known that the temporal patterns of snakebite incidence are associated with snakebite incidence (Chavez et al., 2015 and Ediriweera et al., 2018), but these results are based on national data. Recently, and thanks to the comments, Ediriweera et al., 2021 looked at the first time the spatio-temporal association between snakebite incidence and climatic covariates and showed that this association is not homogeneous inside a country. In our manuscript, we referred to this spatial heterogeneity in the temporal patterns of snakebite incidence, where the association between snakebite dynamics and climate is not homogeneous in the space.

We changed this part of the introduction to clarify that this specific issue (Spatial heterogeneity of temporal patterns of snakebite) is emerging in literature, and we cited the study of Ediriweera et al., 2021.

**Line 67: replace medical care with allopathic medicine (as traditional medicine is also a medical care).  Please add Ediriweera et al (2017), “Health seeking behavior following snakebites in Sri Lanka: Results of an island wide community-based survey” as a reference to support lines 66 – 67.**

Thank you for your suggestion, we changed it and included the reference.

**Lines 98 – 99: a recent study done in Sri Lanka has shown heterogeneity in the relationship between rainfall and snakebite incidence in different climatic zones. Therefore, pls update your statement with the below reference.  Ediriweera et al (2021), “Evaluating spatiotemporal dynamics of snakebite in Sri Lanka: Monthly incidence mapping from a national representative survey sample”.**

Thanks for recommending us this remarkable study. We are sorry for not including this reference in the first draft. After reading this study, we understood that our work is not the first study examining these spatio-temporal effects. Thus, we updated our statement and included this study in the introduction and in the discussion (Lines 119 - 126 and 523, respectively). In addition, we included the difference between our scope and the scope of Ediriweera et al., 2021: We want to apply compartmental models that can be updated to robust epidemiological modeling and help to understand snakebite dynamics theoretically.

**Reviewer #3:**

**General comments:**

**L67: and also because mortality number don’t accurately represent the additional burden caused by physical & mental disabilities following some snakebites**

Thank you for your suggestion. We changed it in the manuscript by including that mortality does not account for that additional burden.

**L81: you previously state that there are problems with extrapolation so you should explain here why your kind of extrapolation is different. For example geographically adjusted, fine scale extrapolation, or something similar that describes why your way of extrapolating is accounting better for the complexity of the situation than what is commonly done (i.e. what you criticized in the last paragraph).**

Thank you for the comment, this difference was not clear. Specifically, we changed in Lines 79 - 86 the statistical extrapolations by their empirical nature, which just find a correlation between risk and covariates; thus, is more difficult to perform predictions and estimation by applying these frameworks. Also, we changed epidemiological modeling by compartmental modeling in all the paper because our model is still not as robust as an epidemiological model (Which also uses venomous snakes’ population dynamics information). In Lines 89 – 94 we also stated the differences between compartmental models and correlational approaches, focusing on the fact that compartmental models can perform prediction easier because they are based on disease’ dynamics rather than on the general equations that statistical and correlational approaches use. Finally, we removed that our modeling approach has never been used in snakebite because of the suggestions made by referee two and your next suggestion (Lines 103 - 104).

**L82-84: I think several authors would argue that they have used mechanistic epidemiological models for estimating temporal patterns in snakebite. Most notably Goldstein et al. 2021 (Integrating human behavior and snake ecology with agent-based models to predict snakebite in high-risk landscapes). In fact I’d be careful calling the models in the current study mechanistic. From my point of view a mechanistic model models specific processes to predict what snakebite incidence should be and then compares the predictions with real data. The current study is a correlative approach, where the correlation between rainfall and incidence is tested. It is partially mechanistic in that it tries to explain the ‘mechanism’ behind snakebite incidence trends (although rainfall alone is a very partial mechanism without accounting for other important drivers of snake activity such as temperature). However, it is not truly mechanistic in the form of a bottom up modelling approach. For this you would have to model how climate should, in theory, effect snake activity and abundance based on their known biology and then test if the predictions actually coincide with incidence. The approach in this paper is great, I’d just be careful with overstating the mechanistic nature of it, especially considering that there are studies in Sri Lanka that similarly correlate rainfall with snakebite incidence (Ediriweera et al. 2016; 2018) and that truly mechanistic snakebite models exist (Goldstein et al. 2021).**

Thank you for the suggestion. We understand that this part was not clear in the manuscript, and it was difficult to understand. Therefore, we restructured this paragraph to emphasize that our model is a compartmental model that assumes the populations as compartments and identifies the flow between these populations based on theoretical equations (i.e., the law of mass action). Thus, each parameter of our compartmental model has biological meaning. We also clarified why these compartmental models are not correlational, because they generate an equation structure representing disease’ dynamics and test how covariates affect specific flows.

In our model, the flow between Susceptible and Envenomed populations represents the incidence. In fact, this flow is the new envenoming cases that occur in a determined timestep. Thus, we represented the incidence by applying the law of mass action on this incidence (See previous study Bravo-Vega et al., 2019) by introducing parameter *β*: The encounter frequency with venomous snakes. Furthermore, we made this parameter dependent on climatic covariates; thus, thanks to our model architecture, we are testing how climatic variables affect this encounter frequency, rather than using a correlational approach that looks for an association between dependent variables and covariates.

**L250-259: I feel like it would have been worth testing the effect of other climate variables, such as e.g. temperature to explain the residual seasonality on top of rainfall driven patterns. Considering that you are trying to provide a mechanistic explanation of snakebite incidence, temperature is an important candidate variable and shouldn’t have been too difficult to include.**

Thank for the valuable appreciation. We included temperature at national data to look at the effect of this variable on snakebite incidence. We tested the association using maximum and minimum temperature, but we did not find any significant effect. Thus, we did not use this variable as a covariate when we performed the analysis at a regional scale because it does not modulate incidence nationally. We changed parts of methodology, results, and discussion to include these new and interesting results.

We also reordered the methodology to increase the coherency to our modeling scheme. First, we start with national data; then we explained how we select our regions to perform the analysis at a regional scale. Then, we explained the models and finally how we adjusted the modeling scheme. Additionally, since we are using different covariates, we cannot compare models using likelihoods. Thus, we used the Akaike Information Criterion to select the best model in each region.

We also changed results: First, we started with national results, where we determined that temperature does not modulate snakebite incidence and that rainfall-driven seasonality is the covariate that modulates snakebite incidence (The best model was Model 4, which we changed this model by the suggestion of referee 2. We eliminated the additional seasonality of the B-splines in this model, and we changed the climatic covariate by the seasonal component of rainfall, which we called rainfall-driven seasonality by a suggestion of referee 1). Then, we show the results of rainfall models for every region. We also changed the whole discussion.

**Minor comments**

**L31: ‘forcing’ is not the best word here, maybe ‘affecting’ or ‘driving’.**

Thank you for your suggestion, we changed it by driving.

**L34: delete ‘other’**

Thank you for your suggestion, we removed this word.

**L50: ‘where’ does not sound right here, do you mean ‘because’. Change typo ‘stablished’ to ‘established’**

Thank you for your suggestion, we rephrased this sentence and removed where. Also, we corrected the typo.

**L53: delete ‘have’**

Thank you for your suggestion, we deleted this word.

**L58: Replace ‘this’ with ‘our’**

Thank you for your suggestion, we changed this word.

**L59: delete ‘it’**

Thank you for your suggestion, we deleted this word.

**L70: change risk’ to risk’s**

Thank you for your suggestion, we changed this word.

**L72: change ‘disease’ monitor tolls’ to ‘disease monitoring tolls’**

Thank you for your suggestion, we changed this word.

**L73: The problem is not the complexity of the extrapolation but the simplification of the extrapolations as compared to the complexity of reality. Reword.**

Thank you for your suggestion. We reworded this sentence by including that performing extrapolation with correlational studies doesn’t account for the reality’ complexity as it was suggested by the other referees.

**L86: change ‘snakes’ species’ to ‘snake species’**

Thank you for your suggestion, we changed this word.

**L86: change ‘which often is poor’ to ‘which is often poor’.**

Thank you for your suggestion, we changed this part of the sentence.

**L86: change ‘In neotropics’ to ‘In the Neotropics’**

Thank you for your suggestion, we changed this part of the sentence.

**L87-88: change ‘which is distributed throughout the south of Mexico to the North of Argentina’ to which is distributed broadly from southern Mexico to northern Argentina’**

Thank you for your suggestion, we changed this sentence.

**L88: delete ‘the’ before envenomings**

Thank you for your suggestion, we removed this word.

**L89-91: too wordy, just say ‘This genus is viviparous with a clutch size of xxxx-xxxx offspring, which are born during the rainy season in many species.’**

Thank you for your suggestion, we included this suggestion, and the sentence became easier to understand.

**L92: change ‘venomous snakes’ populations’ to ‘venomous snake population’**

Thank you for your suggestion, we changed this part of the sentence.

**L94: change ’resulting in an increase 94 in snakebite risk too’ to ‘resulting in an additional increase in snakebite risk’**

Thank you for your suggestion, we changed this part of the sentence.

**L97: delete ‘in both countries’**

Thank you for your suggestion, we deleted this part.

**L98: change ‘pattern’ to ‘patterns’**

Thank you for your suggestion, we changed this word.

**L100: change ‘Colombia becomes’ to ‘Colombia is’**

Thank you for your suggestion, we changed this part of the sentence.

**L101 and throughout: I recommend changing ‘rainfall seasonal patterns’ to ‘seasonal rainfall patterns’**

Thank you for your suggestion, we changed these words.

**L104: delete ‘have’**

Thank you for your suggestion, we removed this word.

**L114: ‘aggregate municipality reported the incidence to reduce its noise’ This needs rewording, I’m not sure what you mean. Do you mean you aggregated individual municipalities’ reported data within each climate region to reduce noise in the data caused by different climate patterns?**

Thank you for your suggestion, we reworded this part of the methodology because it was not clear. We specified that we first used national data to test general associations between climate and incidence. Then, we divided the country in 6 regions with similar rainfall patterns: This had the aim to aggregate municipality data within these regions to reduce noise caused by low occurrence of snakebites and heterogeneous climate patterns. After these changes, the methodology is clearer.

**L116: delete ‘a’ before likelihood**

Thank you for your suggestion, we removed this word.

**L129: change ‘the third model’ to ‘a third model’**

Thank you for your suggestion, we changed this part of the sentence.

**L135-136: change ‘which model fit better the data’ to ‘the model with best fit’**

Thank you for your suggestion, we removed this part because with the new structure of methodology section it was redundant.

**L150 ‘The details and settings that we used to perform this estimation can be seen in S2.’ Is very wordy. You could just add a bracket saying ‘(see S2 for details)’**

Thank you for your comment. In fact, that part was very wordy, so we included your suggestion.

**L152: change ‘likelihood were’ to ‘likelihood was’**

Thank you for your suggestion, we removed this part because with the new structure for methodology it was redundant.

**L161-162: change ‘model 1, model 2, and model 4’ to ‘models 1, 2, and 4’ and change ‘and model 1 and model 3 too’ to ‘and so are models 1 and 3’**

Thank you for your suggestion, we removed this part because with the new structure of methodology section it was redundant.

**L162: please state the version of R you used here**

Thank you for your suggestion, we have included the version of R that we used for each one of the packages.

**L188: capitalize ‘r’, i.e. ‘R environment’ throughout the manuscript**

Thank you for your suggestion, we capitalized this noun.

**L237: change ‘he becomes’ to ‘they become’ (gender neutral)**

Thank you for your suggestion, this was also pointed by another referee. Given that we changed figures and captions, this part was removed.

**L251: change ‘maximizes a likelihood’ to just ‘maximizes likelihood’**

Thank you for your suggestion, we removed this part because it was redundant with the new structure of results section.

**L255: change ‘account’ to ‘accounts’**

Thank you for your suggestion, we removed this part because with the new structure of results section it was redundant.

**L257: change ‘explained better the data’ to ‘explained the data better’**

Thank you for your suggestion, we have changed this part of the sentence.

**L365: change ‘give birth neonates’ to ‘give birth to neonates’**

Thank you for your suggestion, we have modified this part of the sentence.

**L377: wouldn’t floods increase the area shared by snakes and humans, i.e. ring snakes closer to humans?**

Thanks for pointing this out. We did not explain the idea well, so we rephrased this sentence. We have clarified that the floods decrease the not flooded area that snakes and humans share, thus raising the contact between both populations.
