# Supplementary material for: Disentangling snakebite dynamics in Colombia: How does rainfall and temperature drive snakebite temporal patterns?
Source: PLoS Negl Trop Dis. 2022 Mar 31;16(3):e0010270. doi: 10.1371/journal.pntd.0010270 (PMC8970366; doi:10.1371/journal.pntd.0010270)
Supplement: Attachment — Submitted filename: Response to reviewers.docx [file pntd.0010270.s006.docx]

**Manuscript PNTD-D-21-0130: Disentangling snakebite dynamics in Colombia: How does rainfall drive snakebite temporal patterns?**

**Point-by-point response to reviewers’ comments – Lines correspond to manuscript with tracked changes**

**Reviewer #1:**

**1. Overall I am very impressed by how the paper has improved. I only have one minor general comment and related to the same major problem I pointed out before: this study's main limitation hasto be acknowledged from the beginning; the introduction suggests that compartmental models are infalible in comparison with empyrical ones (lines 84-96), whereas the "frequency" of contact estimated in this study is empyrical which means that it has the same limitations of previous studies that estimate incidence. Therefore the introduction should not sell the idea that these models are mechanistic in the same sense that epidemiological models are. In relation to the suggestion made in the abstract about model extrapolation, I think it's extremely risky because overall ecology would not hold outside the Colombian setting, human-snake contact rates also reflect socio-economic aspects of local populations in relation to local snake species and in this case hospital admissions.**

We appreciate the interest of the referee in our work and the constructive comments. Therefore, to make a clearer comparison, we restructured the introduction paragraph that defines the compartmental models and their benefits (L83-97). We recognize that this paragraph was not well written; thus, it was not describing our ideas well. These compartmental models also rely on data and data adjustment; the estimated parameters are also empirical. In fact, without data, this framework became limited, and it could not give information about snakebite epidemiology. Thus, we included this shared limitation with empirical models. On the other hand, compartmental models’ usage is more flexible than empirical models because their structure (Compartments and flows) can be used in other countries, but parameters must be adjusted with new data. In addition, more compartments and flows can be included to represent other socio-economic and ecologic variables that will affect epidemiology.

Given that covariates affect specific flows, the knowledge that compartmental models can generate is more fundamental than empirical models. For example, we know that rainfall modulates snakebite incidence via human-snake contacts, which can be affected by venomous snakes’ abundance, activity, or human exposure by harvesting crops. On the other hand, empirical models determine general associations, where rainfall can modulate incidence, but the specific mechanism behind this association cannot be determined. We also recognize that talking about extrapolation is risky, so we removed it from the article and author summary (L 52-60), and we only included that these models are flexible. After including these modifications, the article is more conservative, and the benefits of compartmental modeling are clearly described, so we want to thank you for your valuable suggestions.

**2. Minor editorial suggestions**

**Line 112 "statistical" instead of "statistic"**

We changed it, Thank you.

**Line 137 Sistema nacional de vigilancia nacional, which is correct, Sistema Nacional de Vigilancia or Sistema de Vigilancia Nacional?**

We revised the name and changed it by Sistema Nacional de Vigilancia en Salud Pública.

**Line 167 Should it be indices instead of indexes?**

We changed it by indices.

**Line 214 We fitted the models**

Changed. Thank you for your suggestion.

**Line 215 We did not use**

Changed. Thank you for your suggestion.

**Line 222 Model XX Fitted data better than model YY**

Changed. Thank you for your suggestion.

**Line 250 After computing the parameter (singular) combinations**

Changed. Thank you for your suggestion.

**Line 254 Suggestion: a between-model threshold of 2 AIC units to determine ...**

Changed. Thank you for your suggestion, it made the sentence clearer.

**Line 264 Is it possible that there is also a role of population growth? The difference between population growth and reports should the attributable effectively to improved reporting, shouldn't it?**

Thank you for noticing it, we included also the effect of population growth as a possible explanation of this increased.

**Line 329 Can be explained in region 6 ....**

Changed. Thank you for your suggestion.

**Line 364 Rainfall seasonal strength ...**

Changed. Thank you for your suggestion.

**Line 366 And does not have a clear temporal, periodic trend?**

Changed. Thank you for your suggestion.

**Line 420 Suggestion : ... more robust models that explicitly use information on snake abundance instead of the mentioned environmental proxies ...**

Changed. Thank you for your suggestion, it made the sentence clearer.
